# Medical Classes in Canada

**Published:** 1860-03

**Authors:** 


MEDICAL CLASSES IN CANADA.—We copy the following statement respecting the number of medical students in Canada, from our contemporary, the *British American Journal* for January, 1860:—

| | |
|---|---|
| University of Victoria College, (Rolph's School, Toronto,) | 64 |
| School of Medicine, Toronto | 56 |
| University of Queen's College, Kingston | 77 |
| University of McGill College, Montreal | 108 |
| School of Medicine, (French Canadian,) Montreal | 62 |
| University of Laval, Quebec | 32 |
| Total | 399 |

